# Development of a predictive model for risk factors of multidrug-resistant bacterial pneumonia in critically ill post-neurosurgical patients

**DOI:** 10.3389/fpubh.2025.1623968

**Published:** 2025-06-25

**Authors:** Aixiang Hu, Dayan Ma, Yanni Lei, Fangqiang Li, Xi Wang, Yuewei Zhang

**Affiliations:** ^1^Infection Control Department, Beijing Tiantan Hospital, Capital Medical University, Beijing, China; ^2^Information Management and Data Center, Beijing Tiantan Hospital, Capital Medical University, Beijing, China; ^3^Department of Critical Care Medicine, Beijing Tiantan Hospital, Capital Medical University, Beijing, China; ^4^Laboratory Diagnosis Center, Beijing Tiantan Hospital, Capital Medical University, Beijing, China

**Keywords:** machine learning models, neurosurgical postoperative care, multidrug-resistant bacterial pneumonia, influencing factors, SHAP interpretability

## Abstract

**Background:**

Machine learning models have emerged as pivotal tools for enhancing the predictive accuracy of multidrug-resistant bacterial pneumonia (MDR-BP) risk in critically ill patients following neurosurgery procedures. By enabling early risk stratification, these models facilitate timely diagnosis and proactive therapeutic interventions. However, existing prediction frameworks exhibit limitations in elucidating the relative importance of risk factors, thereby impeding precise clinical decision-making and individualized patient management.

**Objective:**

To evaluate the performance of six ensemble classification algorithms and three single classification algorithms in predicting MDR-BP risk factors among neurosurgical postoperative critically ill patients, identify the optimal predictive model, and determine key influential factors.

**Methods:**

We conducted a retrospective study involving 750 neurosurgical patients admitted to a neurosurgery center at a tertiary hospital in Beijing between January 2020 and December 2023. Following rigorous data preprocessing, univariate analysis was performed to screen statistically significant variables. The Synthetic Minority Over-sampling Technique (SMOTE) was applied to address class imbalance. Predictive models for MDR-BP risk factors were constructed, and their performance was validated using 10-fold cross-validation to assess mean accuracy, recall, and specificity. The SHapley Additive exPlanations (SHAP) framework was employed to quantify feature importance.

**Results:**

The Random Forest model demonstrated superior performance, achieving the highest mean accuracy (0.775) and AUC value (0.860) compared to other models. SHAP interpretation revealed three critical predictors of MDR-BP: intensive care unit length of stay (ICU-LOS), antibiotic treatment duration, and serum albumin level.

**Conclusion:**

The Random Forest algorithm demonstrates superior predictive accuracy for MDR-BP risk in critically ill post-neurosurgical patients. ICU-LOS, antibiotic treatment duration, and serum albumin level are significant predictors of MDR-BP.

## 1 Introduction

Neurosurgery primarily manages patients with traumatic brain injuries, cranial nerve disorders, and brain tumors, nearly all of whom require surgical intervention. Postoperative neurosurgical patients are highly susceptible to pneumonia due to compromised airway reflexes, neurogenic dysphagia, immunosuppression, prolonged immobilization, and neurogenic pulmonary edema—factors that collectively increase infection risk during recovery. Hospital-acquired pneumonia (HAP) remains a critical challenge in the clinical management of critically ill neurosurgical patients (1). Current studies on HAP in intensive care units (ICU) indicate that most infections are caused by non-fermentative bacteria, which often exhibit high antibiotic resistance, complicating the selection of initial antimicrobial therapy (2). Multidrug-resistant bacterial pneumonia (MDR-BP) in neurosurgical postoperative critically ill patients not only elevates mortality rates among older adult and critically ill populations but also increases economic burdens. Lin et al. (3) reported the the 30 day mortality rate of MDR Acinetobacter calcoaceticus-Acinetobacter baumannii (Acb) complex-associated pneumonia was 31.2% in hospitalized patients. Yang et al. (4) demonstrated that antimicrobial treatment costs for MDR-BP patients with severe cerebral hemorrhage were significantly higher than those for non-MDR-BP cases.

Previous studies have identified several risk factors for *A. baumannii* respiratory tract colonization or infection in ICU patients, including invasive procedures, prolonged ICU stays, mechanical ventilation, enteral feeding, burns, and recent broad-spectrum antibiotic use (5). A study of 3,837 VAP patients revealed that risk factors for *Pseudomonas aeruginosa* (*P. aeruginosa*) VAP in ICU included advanced age, ICU transfers, mechanical ventilation, antibiotic exposure, and admission to units with high *P. aeruginosa* prevalence (6). Although known MDR risk factors encompass prolonged hospitalization before VAP onset, prior antibiotic use, high local antimicrobial resistance rates, immunosuppression, and disease severity, their relative contributions remain unclear, hindering precise algorithmic management (2). Neurosurgical intensive care units (NSICU), specializing in postoperative care for traumatic brain injuries, intracranial tumors, and cerebral hemorrhages, frequently employ invasive interventions such as mechanical ventilation and drainage tubes (1). Whether MDR-BP in NSICU exhibits specialty-specific characteristics warrants investigation. Given these uncertainties, developing precise algorithms for predicting and managing MDR-BP in neurosurgical patients represents a critical public health priority.

Machine learning approaches offer transformative potential in addressing these gaps by enabling robust risk stratification. Recent studies demonstrate that algorithmic models outperform traditional statistical methods in predicting infectious complications (7–12). This study leverages machine learning algorithms to comprehensively predict high-risk features for postoperative MDR-BP in neurosurgical patients, thereby providing evidence-based recommendations for formulating clinical prevention strategies against MDR-BP. Through systematic comparison of six ensemble classification algorithms and three single classification algorithms, we identified the optimal performing machine learning model. Critical features from the time of surgery until the occurrence of MDR-BP were systematically screened and subjected to detailed categorical analysis, enabling iterative model refinement to enhance clinical decision-making precision in infection prevention. The implemented approach ultimately contributes to reducing postoperative MDR-BP incidence, improving patient recovery rates, decreasing healthcare expenditures, and advancing institutional healthcare quality.

The implementation of this research framework not only optimizes MDR-BP prevention protocols within our medical institution but also aligns with broader public health objectives for MDR-BP control across healthcare systems. Methodologically, our feature selection process incorporated temporal dynamics of infection development, while model optimization adopted stratified cross-validation method. The derived predictive framework demonstrates potential for integration into clinical decision support systems, particularly in resource allocation and targeted prophylaxis for high-risk neurosurgical populations.

## 2 Materials and methods

### 2.1 Data collection and ethical considerations

In this retrospective study, data were collected from 750 neurosurgical patients at a tertiary hospital in Beijing between January 2020 and December 2023. Multisource data were extracted from the hospital information system, anesthesia system, electronic medical record system, and hospital-acquired infection surveillance system. The study protocol was approved by the Medical Ethics Committee of Beijing Tiantan Hospital, Capital Medical University (Approval No. KY2022-160-02).

This study included adult patients (≥18 years) who underwent neurosurgical procedures between January 2020 and December 2023, required postoperative NSICU admission for ≥48 h, and subsequently developed pneumonia; exclusion criteria comprised: (1) pre-existing coagulation disorders, (2) pregnancy or lactation status, (3) pneumonia diagnosed prior to NSICU admission, and (4) incomplete medical records (specifically missing NSICU admission/discharge dates, surgical dates, or essential demographic/clinical parameters including sex, age, and body mass index [BMI], calculated as weight in kilograms divided by height in meters squared). The cohort was stratified into two groups based on microbiological confirmation: MDR-BP cases versus non-MDR-BP controls.

#### 2.1.1 Definition of pneumonia

This study included HAP and VAP. HAP was defined as pneumonia occurring ≥48 h after admission without prior invasive mechanical ventilation or active infection at admission. VAP refers to pneumonia that arises more than 48–72 h after endotracheal intubation, including cases occurring within 48 h after extubation or ventilator weaning (13, 14).

#### 2.1.2 Definition of MDR

MDR was defined as non-susceptibility to ≥3 antimicrobial agents across ≥1 antibiotic class (15). Common MDR pathogens in this study, based on institutional laboratory surveillance, included methicillin-resistant staphylococcus aureus (MRSA), carbapenem-resistant *A. baumannii* (CRAB), carbapenem-resistant *Klebsiella pneumoniae* (CRKP), and carbapenem-resistant *P. aeruginosa* (CRPA).

#### 2.1.3 Diagnostic criteria

The diagnosis of pneumonia was established based on both clinical and microbiological criteria, as outlined in the Chinese Guidelines for Diagnosis and Treatment of Hospital-Acquired and Ventilator-Associated Pneumonia in Adults (2018 Edition) (16).

### 2.2 Research methods

#### 2.2.1 Data processing

Following initial screening, 17-dimensional features were selected and categorized into two data types: categorical variables [sex, hypertension, diabetes, preoperative emesis or aspiration (E/A), smoking, gastric tube insertion (GTI), and tracheostomy] processed using one-hot encoding, binary encoding, or ordinal numerical encoding (e.g., “high,” “medium,” “low” mapped to 3, 2, 1); and quantitative variables [age, BMI, surgery duration, ICU length of stay (ICU LOS), ventilator days, corticosteroid therapy duration (CTD), central venous catheter days (CVC days), antibiotic days, glucose level (GLU), serum albumin level (ALB), and procalcitonin level (PCT)] that underwent outlier removal (null values, negatives, non-numeric entries) and min-max normalization. Age and BMI were treated as categorical variables during univariate analysis. Due to missing height/weight data, BMI was available for only 737 samples, establishing this as the effective sample size for BMI-related analyses.

#### 2.2.2 Feature selection

The predictive model was constructed using features selected through univariate analysis. Proper feature selection improves model performance, mitigates overfitting risk, and optimizes training process. Univariate analysis was conducted using SPSS 22.0 to identify predictors of postoperative MDR-BP. Categorical data were expressed as frequencies/percentages and compared using chi-square tests. Normally distributed continuous data were presented as mean ± SD and compared using *t*-test or ANOVA, whereas non-normally distributed data were expressed as median (interquartile range) and analyzed using Mann–Whitney *U* tests. A two-tailed *p*-value <0.05 was considered statistically significant.

#### 2.2.3 Imbalanced data management

In the context of limited data samples (*n* = 737) with imbalanced class distribution (case group: 459, control group: 278), this study implemented the Synthetic Minority Over-sampling Technique (SMOTE) to address class imbalance. The implementation of oversampling techniques on minority class instances significantly improved the model’s classification accuracy for underrepresented categories, thereby enhancing the overall predictive performance of the model.

Following SMOTE processing, the dataset was balanced to yield 918 synthetic samples, comprising 459 instances each from the positive and negative classes.

#### 2.2.4 Predictive model development

A binary classification model was constructed to identify risk/protective factors for postoperative MDR-BP. Multiple algorithms were evaluated: ensemble classifier (Random Forest [RF], AdaBoost, Gradient Boosting, XGBoost, LightGBM, CatBoost) and single classifiers (logistic regression, CART, SVM). Model performance was assessed via 10-fold cross-validation to ensure robustness given limited sample size. The optimal algorithm was selected based on accuracy metrics.

## 3 Results

### 3.1 Variable screening

Patients were stratified into six age groups: <30, 30–39, 40–49, 50–59, 60–69, and ≥70 years. BMI classifications followed the Health Industry Standard of the People’s Republic of China—Criteria of Weight for Adults (WS/T 428–2013): (1) underweight group: BMI < 18.5 kg/m^2^; (2) normal weight group: 18.5 kg/m^2^ ≤ BMI < 24 kg/m^2^; (3) overweight group: 24 kg/m^2^ ≤ BMI < 28 kg/m^2^; (4) obese group: BMI ≥ 28 kg/m^2^.

Univariate analysis revealed significant associations between MDR-BP infection and the following variables: BMI, GTI, tracheostomy, ICU LOS, ventilator days, CTD, CVC days, antibiotic days, GLU, ALB, and PCT (*p* < 0.05). Detailed results are presented in Tables 1, 2.

**Table 1:** Univariate analysis of categorical variables in MDR-BP infection.

Features	Number of people (n)	MDR-BP *n* (%)		Chi-square value	*P-*value
		No	Yes		
Sex				2.153	0.145
Female	305	123 (40.3%)	182 (59.7%)		
Male	445	156 (35.1%)	289 (64.9%)		
Age (years)				3.118	0.684
<30	60	24 (40.0%)	36 (60.0%)		
30–39	108	39 (36.1%)	69 (63.9%)		
40–49	107	33 (30.8%)	74 (69.2%)		
50–59	220	89 (40.5%)	131 (59.5%)		
60–69	187	69 (36.9%)	118 (63.1%)		
≥70	68	25 (36.8%)	43 (63.2%)		
Hypertension				1.159	0.311
No	468	181 (38.7%)	287 (61.3%)		
Yes	282	98 (34.8%)	184 (65.2%)		
Diabetes				1.907	0.177
No	671	244 (36.4%)	427 (63.6%)		
Yes	79	35 (44.3%)	44 (55.7%)		
Smoking				0.014	0.915
No	641	239 (37.3%)	402 (62.7%)		
Yes	109	40 (36.7%)	69 (63.3%)		
BMI				9.250	0.026
<18.5	23	12 (52.2%)	11 (47.8%)		
18.5 ~ 23.9	306	129 (42.2%)	177 (57.8%)		
24 ~ 28	268	84 (31.3%)	184 (68.7%)		
≥28	140	53 (37.9%)	87 (62.1%)		
GTI				52.079	0.000
No	89	64 (71.9%)	25 (28.1%)		
Yes	661	215 (32.5%)	446 (67.5%)		
Tracheostomy				11.469	0.001
No	680	266 (39.1%)	414 (60.9%)		
Yes	70	13 (18.6%)	57 (81.4%)		
E/A					
No	535	333 (62.2%)	202 (37.8%)	0.248	0.619
Yes	215	138 (64.2%)	77 (35.8%)		

Values outside the parentheses indicate case numbers (count), while those within parentheses represent constituent ratios (proportion).

**Table 2:** Univariate analysis of quantitative variables in MDR-BP infection.

Features	MDR-BP *n* (%)		*Z*-value	*P*-value
	No	Yes		
Surgery duration	5.24 (3.75 ~ 6.91)	5.42 (4 ~ 6.98)	−0.929	0.353
ICU LOS	3 (0 ~ 5)	7 (4 ~ 12)	−11.892	0.000
Ventilator days	0 (0 ~ 0)	0 (0 ~ 5)	−8.364	0.000
CTD	2 (0 ~ 5)	4 (2 ~ 8)	−6.839	0.000
CVC days	0 (0 ~ 0)	0 (0 ~ 0)	−3.932	0.000
Antibiotic days	2 (0 ~ 5)	8 (3 ~ 15)	−11.551	0.000
GLU	11.96 (9.89 ~ 15.89)	13.38 (10.6 ~ 16.41)	−3.210	0.001
ALB	31.6 (28.65 ~ 34.60)	29.5 (26.85 ~ 32.20)	−6.365	0.000
PCT	0.275 (0.1 ~ 0.72)	0.419 (0.20 ~ 1.43)	−4.792	0.000

Values outside the parentheses indicate the median, while those within parentheses represent the first and third quartiles (Q1–Q3).

### 3.2 Model construction and comparison

Based on the results presented in Table 3, the mean accuracy (ACC), mean recall, and mean specificity derived from 10-fold cross-validation were comparatively analyzed across the nine models. The analysis revealed that all models exhibited notably low specificity. This phenomenon may be attributed to class imbalance in the dataset and inherent model bias toward the positive class. To address these limitations, subsequent optimizations will focus on class distribution imbalance mitigation and hyperparameter tuning to enhance model generalizability and performance robustness.

**Table 3:** Comparative analysis of evaluation metrics across classification algorithms.

Evaluation metrics	Ensemble classification algorithms						Single classification algorithms		
	RF	AdaBoost	GBDT	XGBoost	LightGBM	CatBoost	Logistic	CART	SVC
ACC	0.703	0.703	0.689	0.688	0.674	0.716	0.722	0.685	0.720
Recall	0.819	0.802	0.802	0.786	0.782	0.838	0.842	0.683	0.837
Specificity	0.533	0.560	0.524	0.534	0.509	0.529	0.553	0.565	0.554

#### 3.2.1 Class imbalance handling and model optimization

Given the limited dataset (737 cases) with imbalanced class distribution (positive: 459, negative: 278), this study implemented the SMOTE to address class imbalance. The oversampling methodology enhanced the model’s capacity to identify minority class patterns, thereby improving overall model performance.

Following SMOTE processing, the dataset was expanded to 918 balanced samples (459 positive and 459 negative samples). The performance metrics of various models on the oversampled dataset are presented in Table 4. Comparative analysis revealed that model specificity metrics showed systematic improvement after class balancing, highlighting that inherent class imbalance degrades classification efficacy for minority patterns and consequently compromises overall model performance. Notably, the non-optimized RF model achieved superior performance with an accuracy of 0.775, demonstrating significant improvement over Logistic Regression’s performance on the original imbalanced dataset.

**Table 4:** Comparative evaluation of performance metrics for oversampled datasets across diverse classification algorithmic models.

Evaluation metrics	Ensemble classification algorithms						Single classification algorithms		
	RF	AdaBoost	GBDT	XGBoost	LightGBM	CatBoost	Logistic	CART	SVC
ACC	0.775	0.721	0.721	0.745	0.750	0.760	0.686	0.686	0.691
Recall	0.710	0.667	0.679	0.687	0.683	0.705	0.600	0.534	0.604
Specificity	0.755	0.734	0.699	0.715	0.747	0.750	0.764	0.670	0.767

#### 3.2.2 Model hyperparameter tuning

As an ensemble learning algorithm, the performance of the RF model is highly dependent on its hyperparameter configuration. Systematic hyperparameter tuning can significantly enhance model accuracy. Through exhaustive grid search of all critical parameter combinations for the RF model, we identified the optimal parameter set {“n_estimators”:190,“min_samples_leaf”:1, “min_samples_split”:2,“max_features”: “sqrt,”“criterion”:“gini”}, achieving a 10-fold cross-validation mean accuracy of 0.782.

**Figure 1:**
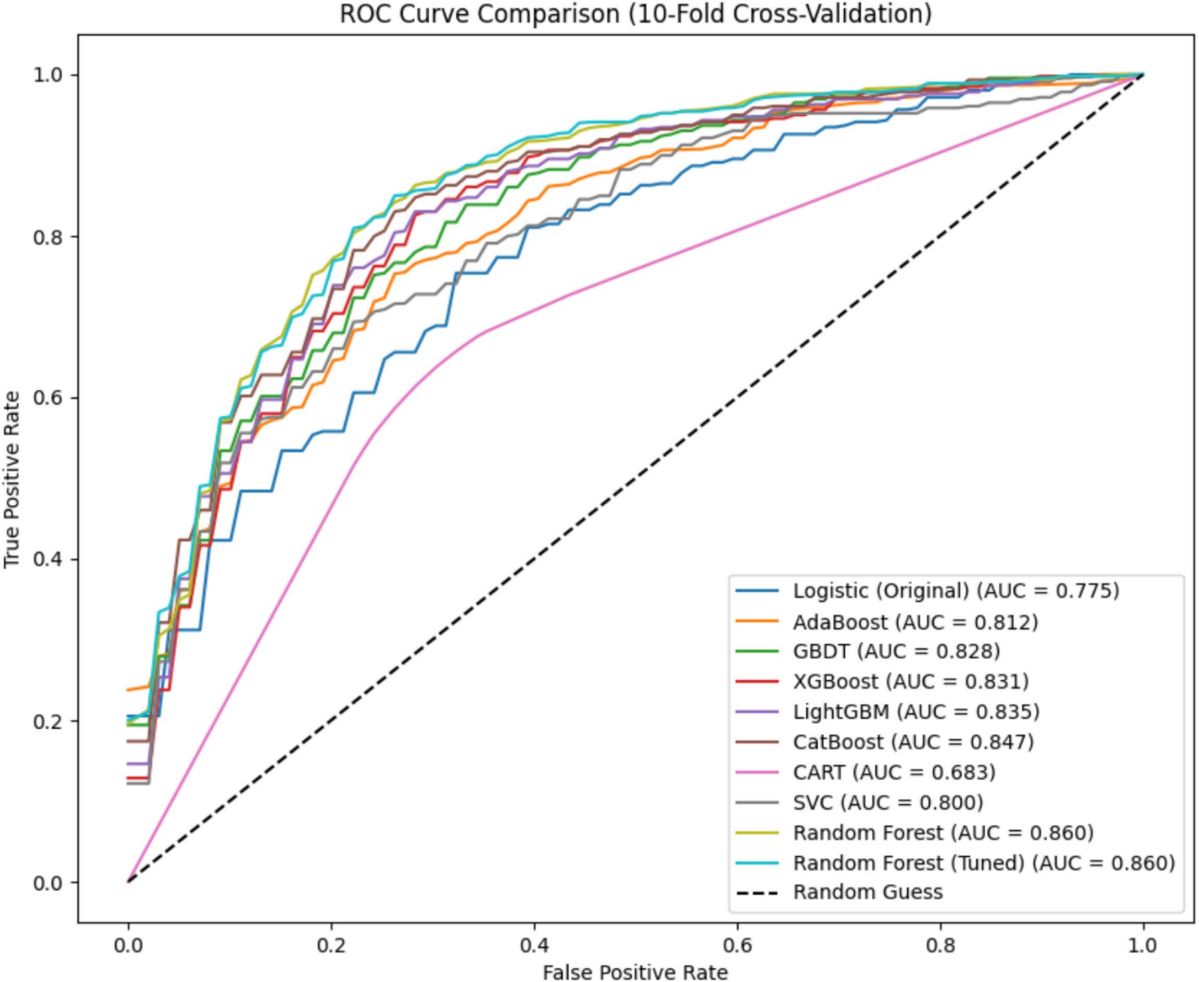
Comparative analysis of ROC curves pre- and post-hyperparameter optimization.

Following the construction of postoperative MDR-BP in neurosurgical patients risk prediction model, we conducted a comparative analysis of Receiver Operating Characteristic (ROC) curves across different models. Notably, the logistic regression model was trained and evaluated on the original dataset, whereas both pre-optimized and post-optimized RF models along with other classification algorithms (without hyperparameter adjustment) were trained and assessed using the oversampled dataset. The average ROC curves for all evaluated models are presented in Figure 1.

The RF model achieved a significantly larger Area Under the Curve (AUC) compared to other models. Notably, its AUC value substantially surpassed that of the logistic regression model on the original dataset, suggesting that imbalanced class distribution mitigation can enhance overall model performance to some extent. Remarkably, the RF model approached its performance ceiling even without hyperparameter tuning, indicating limited potential for further performance enhancement through parameter optimization. Furthermore, all AUC values were calculated as the mean of 10-fold cross-validation outcomes, ensuring data reliability and methodological stability.

### 3.3 Key predictive variables in the model

**Figure 2:**
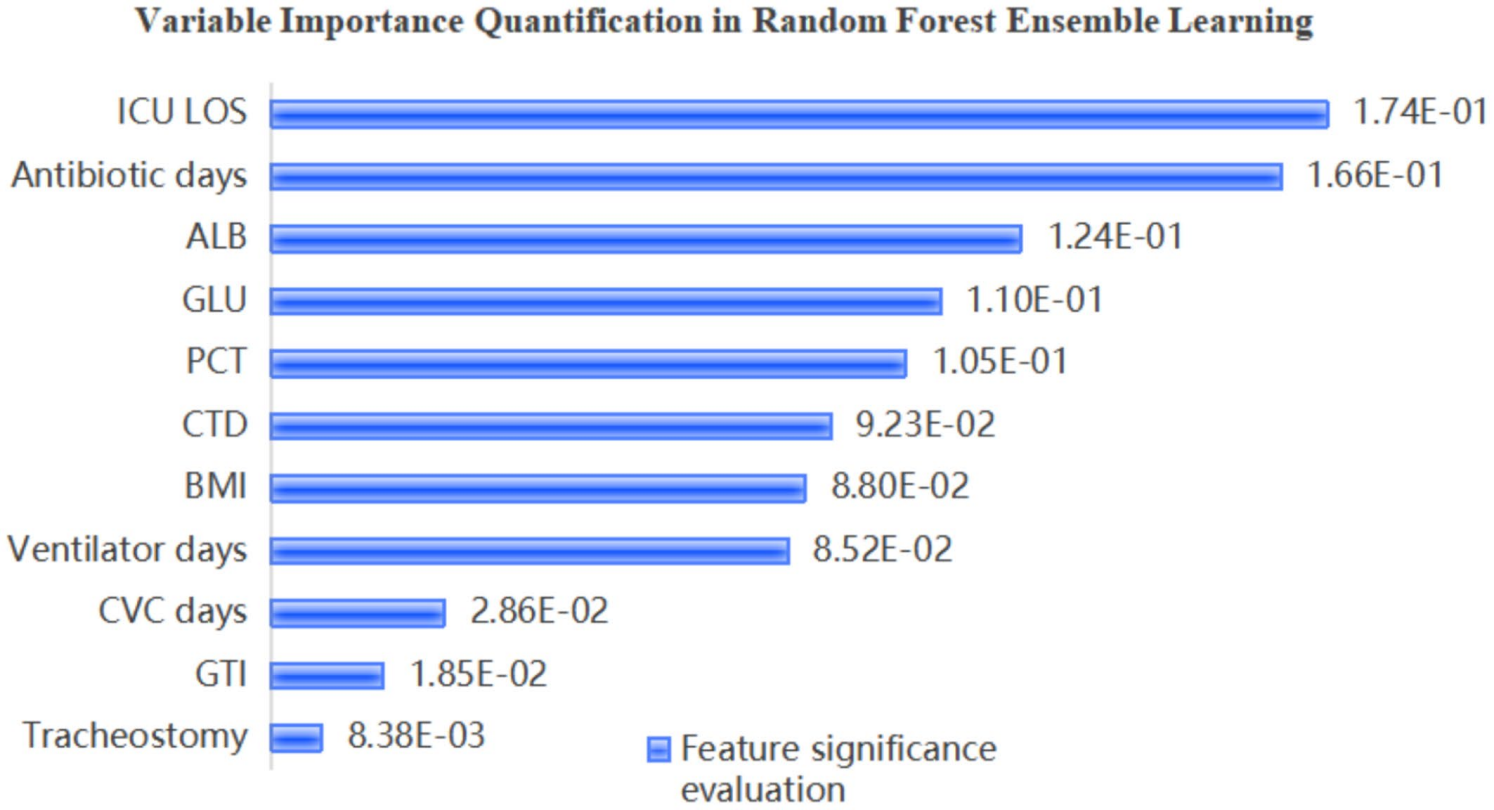
Variable importance quantification in random forest ensemble learning.

Identifying the influential factors affecting MDR-BP risk is critical for guiding clinical prioritization of high-impact variables and formulating targeted prevention and control strategies. The feature importance rankings derived from the optimized RF model are demonstrated in Figure 2. The analysis revealed that ICU LOS, antibiotic days, ALB, and GLU were significant predictors of MDR pneumonia.

**Figure 3:**
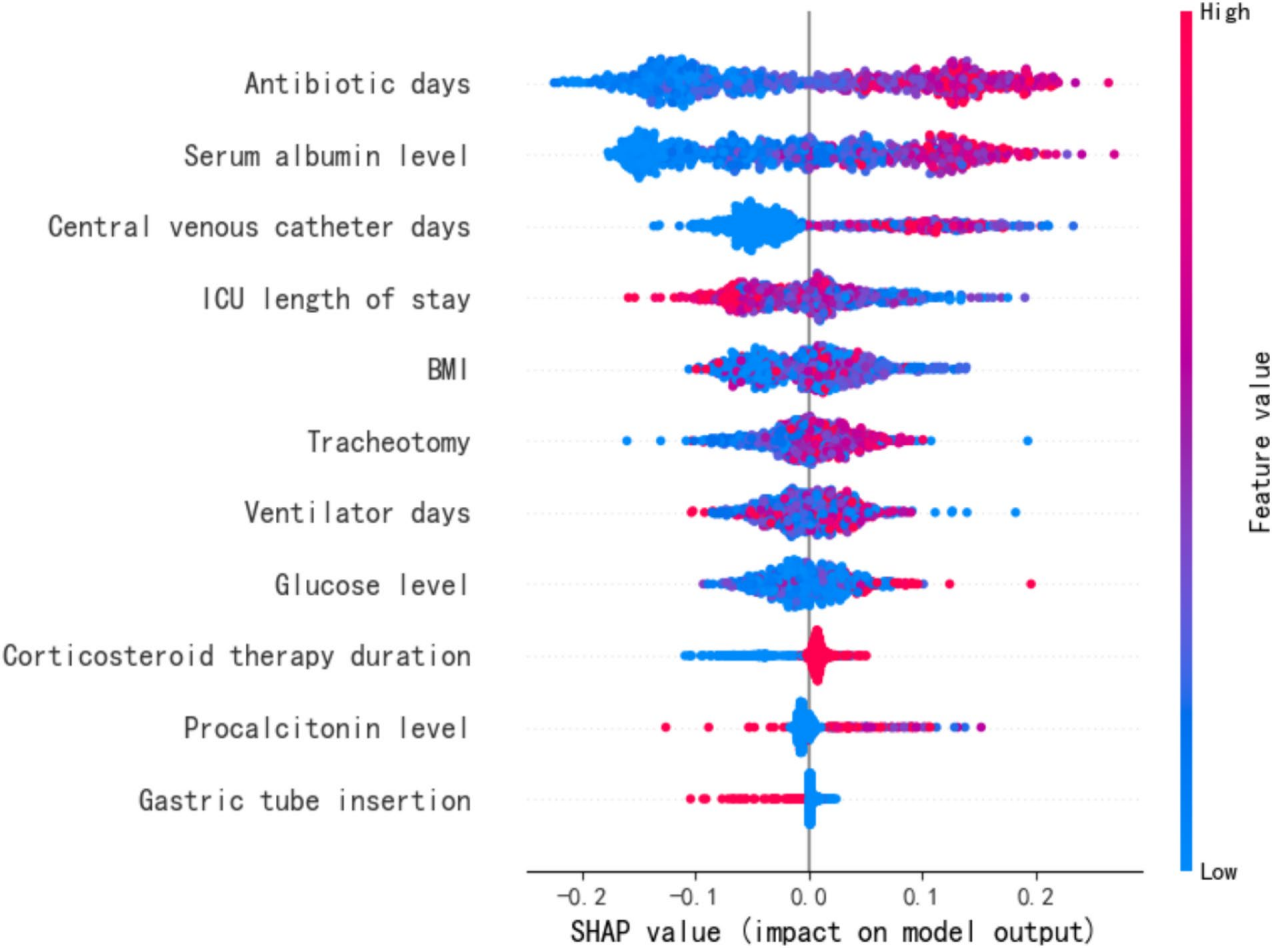
SHAP values for RF model.

The SHapley Additive exPlanations (SHAP) method was employed to interpret the feature importance of the Random Forest (RF) model in predicting MDR-BP, as illustrated in Figure 3. SHAP values computed by the RF model on the dataset served as global feature importance metrics, characterizing the directional influence (positive or negative) of features on predicting the positive class (MDR-BP patients). Features were ranked in descending order based on the absolute values of their SHAP scores.

The results demonstrated that the strongest predictors in the RF model included antibiotic days, ALB, CVC days, and ICU LOS. Notably, ALB emerged as a negative predictor of infection. Comprehensive analysis revealed that the importance scores of GLU and CVC days exhibited high variability, whereas ICU LOS, antibiotic days, and ALB displayed relatively stable predictive performance. These findings identify the latter three variables as critical and robust indicators for MDR pneumonia prediction.

## 4 Discussion

This study identified ICU LOS, duration of antibiotic therapy, and serum ALB as critical predictive variables for MDR-BP in neurosurgical critically ill patients using an optimized machine learning approach. Neurosurgical critically ill patients demonstrate substantially increased infection risks due to acquired immunosuppression resulting from surgical trauma, administration of corticosteroid or barbiturate, or primary brain injury. HAP represents a major complication in the NSICU, with prolonged ICU LOS and extended use of broad-spectrum antibiotics synergistically increasing the likelihood of MDR-BP development (1, 5, 17, 18). MDR-BP complicates clinical management, prolongs hospitalization, elevates mortality rates, and escalates direct healthcare costs (1, 19). In NSICU settings, MDR-BP presents a clinically challenging scenario, necessitating stringent environmental disinfection protocols and rigorous implementation of infection prevention and control measures to mitigate cross-contamination risks. Early prediction of individualized risk factors for MDR-BP could enable prioritized prophylactic interventions for high-risk patients, consequently decreasing infection rates and enhancing treatment outcomes (6).

Traditional statistical inference models depend on predetermined assumptions regarding data distributions and fixed model structures (e.g., linear or polynomial frameworks), demonstrating restricted capacity to identify complex non-linear relationships. The emergence of big data analytics and enhanced computational power has facilitated superior predictive performance through machine learning methodologies (7). Machine learning algorithms, unconstrained by strict distributional assumptions, excel at pattern recognition through complex architectures (e.g., neural networks, tree-based models) capable of modeling non-linear interactions, collinear effects, and high-dimensional feature spaces. Prior studies have demonstrated the superior discriminative accuracy of machine learning models (8–12). The individual contributing factors for MDR-BP in neurosurgical critically ill patients admitted to the NICU are complex and dynamic, with potential collinear interactions among variables. By leveraging advanced machine learning algorithms to systematically analyze and compare these multidimensional risk parameters, we intend to identify high-risk predictive variables with significant weighting coefficients. This analytical approach will facilitate the establishment of an early warning system for implementing targeted prevention and control strategies against MDR-BP in neurocritical care settings. To identify the optimal predictive model for our dataset, we systematically compared six ensemble classification algorithms against three single-algorithm classifiers.

Due to class imbalance in the dataset, the models exhibited significant bias toward the positive class, resulting in suboptimal specificity across all nine models when tested on the original imbalanced dataset. In clinical practice, neurosurgical critical care patients typically require prolonged hospitalization in the NSICU, where the incidence of MDR-BP substantially exceeds that of non-MDR-BP (2). To mitigate this data imbalance and improve model performance, we implemented the SMOTE oversampling methodology. After applying oversampling techniques to the dataset, all models demonstrated measurable enhancements in specificity metrics. Notably, the unoptimized RF model achieved superior performance with an accuracy of 0.775 and specificity of 0.755, significantly outperforming the logistic regression model’s baseline accuracy of 0.722 on the original dataset. Subsequent hyperparameter optimization of the RF model yielded a mean accuracy of 0.782 through 10-fold cross-validation. For comprehensive performance comparison, ROC curve analysis revealed that both pre-optimization and post-optimization RF models attained maximal AUC values of 0.86 in the oversampled dataset, establishing their superior predictive performance compared to alternative models.

The feature importance analysis from the optimized RF model identified ICU LOS, antibiotic days, ALB, and GLU as significant predictors of MDR-BP. Subsequent SHAP value calculations yielded the following global feature importance ranking: antibiotic days, ALB, CVC days, and ICU LOS. Notably, ALB consistently demonstrated a negative correlation with infection risk. While GLU and CVC days showed substantial variability in predictive contributions, ICU LOS, antibiotic days, and ALB maintained relatively stable predictive performance across analyses, confirming their status as predictors of MDR-BP development.

Prolonged ICU hospitalization was identified as a risk factor for MDR-BP. Extended ICU stays correlate with greater exposure to invasive procedures and MDR, consequently elevating infection risk (5). Similarly, prolonged antibiotic use prior to infection establishment intensifies antimicrobial selection pressure, promoting MDR pathogen proliferation and correspondingly elevating MDR-BP susceptibility. This underscores the imperative for antimicrobial stewardship programs to minimize unnecessary antibiotic exposure (13). Another feature associated with MDR-BP identified by the predictive model was ALB. The association between ALB and MDR-BP risk following neurosurgical procedures may be mediated through multiple pathophysiological mechanisms, with one proposed mechanism involving albumin’s immunomodulatory capacity to potentiate immune cell activation (20). Hypoalbuminemia may exacerbate the immunosuppressive state through compromised immunostimulatory function, thereby further elevating pneumonia susceptibility by impairing antimicrobial defense mechanisms (21). The second mechanism is mediated by the antioxidant and anti-inflammatory properties of serum proteins (21, 22). Hypoalbuminemia may elevate the risk of postoperative pneumonia through its failure to adequately constrain neuroinflammatory responses following neurosurgical procedures. Under physiological conditions, these regulatory responses stimulate tissue repair mechanisms and mitigate cellular necrosis; however, when dysregulated or excessive, sustained inflammatory cascades and oxidative stress can precipitate secondary injury, thereby amplifying patient susceptibility to nosocomial infections (21, 23).

This study’s limitations include its constrained sample size potentially impacting model performance, necessitating future validation with larger datasets, along with prospective studies to systematically identify MDR-BP determinants. While the current investigation primarily focused on patient-specific factors, future evaluations should comprehensively assess potential interactions with environmental variables, particularly regarding critical procedural factors requiring rigorous examination: compliance with aseptic protocols during invasive interventions (e.g., tracheostomy), standardization of daily endotracheal tube care practices, and other nosocomial infection control measures. These operational parameters necessitate quantitative assessment and quality optimization through well-designed prospective observational studies.

## 5 Conclusion

This study systematically evaluated the predictive performance of six ensemble classifiers and three single classifiers for postoperative MDR-BP in neurosurgical populations, with the RF model demonstrating significantly superior discriminative accuracy among all tested algorithms. SHAP value analysis of the optimal RF model validated three key clinical predictors: ICU LOS, antibiotic therapy duration, and serum ALB levels. To enhance clinical translation, future researches should: (1) conduct prospective observational studies to further verify these risk factors, (2) incorporate dynamic process-related variables into predictive models, and (3) optimize model structure to improve accuracy, ultimately developing a clinically applicable decision-support system for MDR-BP prevention in neurosurgical practice. It should be noted that as a single-center pilot investigation, these findings should undergo protocol refinement before gradual implementation across secondary and primary healthcare institutions, with the long-term goal of informing evidence-based public health policies for reducing postoperative MDR-BP incidence in critically ill neurosurgical patients.

## Data Availability

The raw data supporting the conclusions of this article will be made available by the authors, without undue reservation.
